# Editorial: New insights into oxidative medicine: unraveling the complexity of oxidative stress in health and disease

**DOI:** 10.3389/fmolb.2025.1743079

**Published:** 2025-11-27

**Authors:** Claudia Fiorillo, Amir M. Alsharabasy, Matteo Becatti

**Affiliations:** 1 Department of Experimental and Clinical Biomedical Sciences “Mario Serio”, University of Firenze, Firenze, Italy; 2 CÚRAM, Research Ireland Centre for Medical Devices, University of Galway, Galway, Ireland

**Keywords:** oxidative stress, oxidative medicine, redox balance, redox status, reactive oxygen species, lifestyle

One of the most common molecular mechanisms affecting health and disease is oxidative stress (OS)- a condition arising from an imbalance between oxidant production and antioxidant defenses-which contributes to inflammation, metabolic alterations, tissue damage, and aging. The ten original and review articles in The Research Topic *“New Insights into Oxidative Medicine: Unraveling the Complexity of Oxidative Stress in Health and Disease”* collectively highlight the multifaceted nature of oxidative processes and their clinical implications, spanning from molecular biology to population-level epidemiology.

Several studies contained in this Research Topic explored how oxidative balance is related to lifestyle and metabolic status. Using large-scale data from the National Health and Nutrition Examination Survey (NHANES), Zhang et al. demonstrated that an increased oxidative balance score (OBS), integrating dietary and behavioral factors, inversely correlates with the risk of rheumatoid arthritis, suggesting that prevention could be achieved by modulating redox status through nutrition and lifestyle.

Similarly, in an epidemiological study, Tao et al. found a negative association between OBS and hypertension, particularly in younger adults, thus emphasizing the protective role of antioxidants against cardiovascular risk. These findings were reinforced by Qin et al., which introduced the novel *Life’s Crucial 9* (LC9) score, integrating mental health as a component of cardiovascular wellbeing, and showed that systemic inflammation and oxidative stress mediate, at least in part, its association with chronic kidney disease, emphasizing the holistic importance of psychosomatic and redox homeostasis in systemic disorders.

Oxidative mechanisms were further explored, through innovative experimental models, at the molecular and cellular level, in the study by Cao et al. who investigated the effects of single-wall carbon nanohorns on nasopharyngeal carcinoma cells and identified endoplasmic reticulum stress as the key mediator of ROS-induced apoptosis, offering valuable insights into the safety and potential biomedical applications of nanomaterials. In the study by Wu et al. a comprehensive analysis of oxidative stress-related genes in uveal melanoma and the prognostic value of *CALM1*, a crucial modulator of apoptosis and antioxidant defense are reported. These findings illustrate how dysregulated oxidative mechanisms may drive both degenerative and proliferative diseases, depending on cellular context and adaptive capacity.

Both the studies of Zhao et al. and Luo et al. clearly show how redox status and immune function are strictly linked. Mitochondrial dysfunction has been reported by Zhao et al. in systemic lupus erythematosus (SLE), highlighting the promising diagnostic value of mitochondrial mass and membrane potential in lymphocytes as biomarkers of disease activity. In parallel, Luo et al. reviewed oxidative stress markers and antioxidants in atopic dermatitis, emphasizing how reactive oxygen species have a role in skin barrier dysfunction and chronic inflammation, and describing emerging antioxidant delivery systems such as nanomaterials, hydrogels, and microneedles. These findings collectively demonstrate that ROS represent central mediators at the interface between immunity, metabolism, and tissue integrity.

Expanding the systemic view, Nuñez-Selles et al. offered a comprehensive review linking oxidative biomarkers to the progression of hypertension and diabetes mellitus, indicating OS as both an inducer and amplifier of cardiometabolic dysfunction. The authors propose the concept of “precision redox medicine” where tailored antioxidant treatment and lifestyle modification are managed by specific biomarker levels. Zhu et al., in their article, contributed to this translational perspective by developing and validating a predictive model for Meige syndrome based on redox markers, where albumin, gamma-glutamyl transferase, total bilirubin, and the urea nitrogen-to-creatinine ratio represent independent predictors. In another study, Mao et al. connect oxidative stress to immune senescence and disease severity and highlight its potential as a therapeutic target in community-acquired pneumonia in older adults.

Taken together, in the articles included in this Research Topic, oxidative stress is proposed as a unifying mechanism across diverse diseases, establishing connections among cellular signaling, metabolism, and systemic physiology. They also illustrate the transition of oxidative medicine from a descriptive field to one supported by quantitative biomarkers, predictive modeling, and mechanistic insight. The convergence of epidemiology, molecular biology, and translational research showed here highlight the growing potential of redox biology to drive preventive strategies and personalized therapies.

In conclusion, this Research Topic demonstrates that oxidative stress must not be merely considered in terms of pathological by-products but a central regulator of cellular adaptation. Continued interdisciplinary research integrating omics technologies, computational modeling, and clinical validation will be crucial to translate redox science into targeted interventions for human health. The editors gratefully acknowledge the contribution of all authors, reviewers, and collaborators whose work has advanced our collective understanding of oxidative medicine.

